# Research progress on predicting KRAS gene mutations in colorectal cancer by combining radiomics and multimodal medical imaging

**DOI:** 10.3389/fonc.2025.1605915

**Published:** 2025-08-20

**Authors:** Min Ai, Li Li, Shimei Fan, Cheng He, Yi Guo, Yang He

**Affiliations:** ^1^ Department of Anesthesiology, Nanan District People’s Hospital of Chongqing, Chongqing, China; ^2^ Pathology Department, Chongqing Emergency Medical Center, Chongqing University Central Hospital, Chongqing Key Laboratory of Emergency Medicine, Chongqing, China; ^3^ Physical Examination Center, Chongqing Emergency Medical Center, Chongqing University Central Hospital, Chongqing Key Laboratory of Emergency Medicine, Chongqing, China; ^4^ Medical Imaging Department, Chongqing Emergency Medical Center, Chongqing University Central Hospital, Chongqing Key Laboratory of Emergency Medicine, Chongqing, China; ^5^ Department of Radiology, Guang an District People’s Hospital of Sichuan Province, Guang an, Sichuan, China

**Keywords:** colorectal cancer, KRAS, radiomics, multimodal medical imaging, predicting

## Abstract

Colorectal cancer (CRC), a highly prevalent malignant tumor in clinical practice, poses a serious threat to human health. In 2015, the relevant guidelines issued by the United States clearly stipulated that only patients with the wild-type kirsten rat sarcoma viral oncogene homologue (KRAS) gene were recommended to receive epidermal growth factor receptor (EGFR) inhibitor treatment. Therefore, accurately predicting the status of the KRAS gene plays a crucial role in formulating scientific and reasonable treatment plans and improving prognosis. Currently, multimodal medical imaging techniques, such as computed tomography (CT), magnetic resonance imaging (MRI), and positron emission tomography (PET), which rely on their respective advantages, have been widely applied in the preoperative evaluation of CRC and have become essential examination methods for the diagnosis of CRC. Radiomics was proposed by Lambin in 2012. This technology can extract features of medical images in a High throughput manner and conduct a quantitative analysis of the pathophysiological changes in lesions. In recent years, the integration of multimodal medical imaging and radiomics technology has opened a new path for predicting the mutation status of the KRAS gene and has achieved fruitful results. This article systematically reviews the research progress of radiomics and multimodal medical imaging in predicting CRC related gene mutations, deeply analyses the predictive efficiency of different imaging techniques and feature extraction methods for CRC related gene mutations, and aims to promote the transformation of scientific research achievements into clinical practice, providing a scientific and solid theoretical basis for clinicians to formulate precise treatment plans.

## 1 Introduction

Colorectal cancer (CRC), a highly prevalent malignant tumor of the digestive system, ranks among the top cancers in terms of global incidence and mortality rates, accounting for 10% of new cancer cases and cancer related deaths (1). It poses a serious threat to human health. Research has shown that the mutation statuses of genes such as KRAS and BRAF, which are closely associated with CRC, directly impact the treatment plans and prognosis of patients (2, 3). Since 2015, the National Comprehensive Cancer Network (NCCN) guidelines in the United States have clearly recommended that tumor tissue KRAS mutation genotyping should be carried out for all suspected or confirmed metastatic CRC patients (4). The core reason that the NCCN Guidelines designate KRAS as a decision point for EGFR inhibitor therapy in CRC patients lies in its strong predictive value for treatment efficacy. As a key downstream molecule in the EGFR pathway, mutant KRAS (KRAS MUT) continuously activates signal transduction, leading to the innate resistance of tumors to EGFR inhibitors. By detecting the KRAS status, clinicians can accurately identify populations likely to benefit, maximizing the effectiveness of treatment. These findings provide a scientific basis for personalized medicine and lay a foundation for the development of subsequent targeted drugs and combination therapy strategies. Moreover, the KRAS gene is not only one of the most critical mutated genes in CRC (5), but also deeply involved in the signal transduction of EGFR, influencing the clinical course of CRC across multiple dimensions including pathogenesis, progression, clinical treatment, and patient prognosis (6).

In clinical treatment, the mutation status of the KRAS gene is a key determinant of the therapeutic effect of anti EGFR monoclonal antibody therapy (7). Research indicates that more than 90% of patients with KRAS mutant CRC are resistant to molecularly targeted drugs such as cetuximab (8). In contrast, advanced CRC patients with wild type KRAS can benefit significantly from anti EGFR monoclonal antibody therapy. In particular, for patients with left sided CRC, the combination of chemotherapy and targeted therapy can significantly prolong overall survival (9). In addition, the effect of preoperative chemotherapy in patients with KRAS mutation is significantly inferior to that in patients with wild type KRAS, and the overall survival rate of these patients is relatively low. Clinical data show that approximately 40% of CRC patients have KRAS mutations (10). This characteristic is crucial for predicting patients’ responses to anti EGFR antibody therapy, as mutant patients often respond poorly to such treatments (11).

Although histopathological examination is currently the gold standard for gene detection, it has significant drawbacks (12). The detection process is not only invasive and time consuming but also poses a risk of tumor dissemination. Moreover, the heterogeneity of tumor tissues can affect the representativeness of sampling, limiting the accuracy of test results (13). Owing to its advantages of being noninvasive and enabling real time monitoring, liquid biopsy has shown potential in predicting KRAS gene mutations in CRC. However, liquid biopsy has problems such as low sensitivity, limited biopsy range, and lack of unified standards, which restrict its clinical promotion. In view of this, in recent years, whether noninvasive and holistic imaging modalities can be leveraged to determine CRC genotypes, specifically to assess preoperative KRAS mutation status in CRC patients, has become a focus of intensive investigation in recent years.

In recent years, multimodal medical imaging techniques such as CT, MRI, and PET have been widely used in the preoperative assessment of CRC patients and have achieved satisfactory results in predicting the KRAS gene mutation status (14). As an emerging technology in the medical field, radiomics can extract abundant feature information from multimodal medical images such as CT, MRI, and PET images in a high throughput manner and can be used to quantitatively separate subtle structural changes within tumors (15). It has gradually been applied to multiple clinical aspects of CRC patients, including preoperative diagnosis, treatment planning, and prognosis assessment (16, 17). By exploring the potential relationship between radiomics features and gene mutations in CRC, clinicians can use multimodal medical imaging to predict whether patients carry specific gene mutations, providing key evidence for subsequent personalized treatment (18).

This study systematically reviews research (2002-2024) on KRAS mutation prediction in CRC using multimodal imaging and radiomics. We compare machine learning algorithms, feature selection strategies, and validation metrics, with particular focus on deep learning versus conventional radiomics models. We analyze relationships between imaging technologies (CT, MRI, PET) and KRAS mutations, summarize existing research, and examine radiomics features’ role in prediction models.

## 2 Survey methodology

This study employed a systematic literature search methodology following the PRISMA guidelines. The search encompassed English language publications from January 2002 to December 2024 across four major databases: pubMed, wweb of science, sciencedirect, and springerLink. A comprehensive search strategy combining medical subject headings (MeSH) terms and free text keywords was implemented, with the core search query being (“colorectal cancer” OR “CRC”) AND (“KRAS” OR “Kirsten rat sarcoma viral oncogene homologue”) AND (“radiomics” OR “texture analysis” OR “imaging biomarkers” OR “CT” OR “MRI” OR “PET” OR “multimodal imaging”). The initial search yielded 258 publications that underwent hierarchical screening on the following criteria: (1) Preliminary screening criteria: study type: original research or meta analysis; sample size: ≥20 cases; documentation of specific KRAS mutation detection methods; (2) refined screening criteria: detailed description of radiomics feature extraction methodology; reported model validation metrics (AUC, sensitivity, etc.); and specification of imaging acquisition parameters. After duplicate removal using EndNote X9, two independent researchers screened titles and abstracts, with any discrepancies resolved by a third reviewer (**Table 1**). Through multidimensional analysis, this study aimed to systematically investigate the critical scientific challenges in predicting the KRAS mutation status in patients with CRC using radiomics features and multimodal medical imaging technologies, and seeks to provide theoretical support for optimizing early clinical diagnosis protocols and developing personalized treatment strategies for CRC patients, with the ultimate goal of improving clinical outcomes.

**Table 1:** Previously reported models for predicting KRAS gene mutations in colorectal cancer based on radiomics features and multimodal medical imaging.

	Authors/Years	Paper title	Data type and main radiomics parameters	AUC (95%CI)	Accuracy	Sensitivity	Specificity	Main conclusions
1	Gan Y, Hu Q, Shen Q/2025 (19)	Comparison of Intratumoral and Peritumoral Deep Learning, Radiomics, and Fusion Models for Predicting KRAS Gene Mutations in Rectal Cancer Based on Endorectal Ultrasound Imaging	ultrasound images Using radiomics, we identified 15, 11, 30, and 18 key features from a pool of 1032 features extracted from the original ROI and extended 10-pixel, 20-pixel, and 30-pixel patches, respectively	0.896 (0.8304–0.9608)	0.846	0.88	0.805	The feature-based fusion model DLRex-pand10_FB can be employed to predict KRAS gene muta-tions based on pretreatment endorectal ultrasound images of rectal cancer.
2	Zhao H, Su Y, Wang Y/2024 (20)	Using tumor habitat-derived radiomic analysis during pretreatment 18F-FDG PET for predicting KRAS/NRAS/BRAF mutations in colorectal cancer. Cancer Imaging	PET/CT seven features from low_metabolism_habitat, 12 from high_metabolism_habitat, and 11 from the peritumoral region	0.759 (CI:0.585-0.909)	0.757	0.810	0.688	The habitat-derived radiomic features were found to be helpful in stratifying the status of KRAS/NRAS/BRAF in CRC patients.
3	Xiang Y, Li S, Song M/2023 (21)	KRAS status predicted by pretreatment MRI radiomics was associated with lung metastasis in locally advanced rectal cancer patients	48 wavelet features, 42 texture features, 540 histograms of oriented gradient (HOG) features, and 156 statistical features	0.983	–	-0.85	0.82	We established and validated a radiomic model for predicting KRAS status in LARC. Patients with high RS experienced more lung metastases.
4	Ricci Lara MA, Esposito MI, Aineseder M / 2023 (22)	Radiomics and Machine Learning for prediction of two-year disease-specific mortality and KRAS mutation status in metastatic colorectal cancer	portal venous phase CT Features can be classified into three groups: (1) first-order or histogram features; (2) shape features; (3) texture features, calculated with the gray level cooccurrence matrix (GLCM), gray level size zone matrix (GLSZM), gray level run length matrix (GLRLM) and gray level dependence matrix (GLDM)	0.905 (0.762-0.983)	0.878	0.873	0.823	Predicting the prognosis of patients with metastatic colorectal cancer is useful for making timely decisions about the best treatment options.
5	Cao Y, Zhang J, Huang L / 2023 (18)	Construction of prediction model for KRAS mutation status of colorectal cancer based on CT radiomics	triphasic enhanced CT Four arterial phase (AP), three venous phase (VP), and seven delayed phase (DP) radiomics features were retained as the final signatures for predicting KRAS mutations.	0.772 (0.720–0.823)	0.716	0.792	0..646	The clinical-radiomics fusion model has the best predictive performance for predicting the mutation status of KRAS in CRC.
6	Alshuhri MS, Alduhyyim A, Al-Mubarak H, Alhulail AA / 2023 (23)	Investigating the Feasibility of Predicting KRAS Status, Tumor Staging, and Extramural Venous Invasion in Colorectal Cancer Using Inter-Platform Magnetic Resonance Imaging Radiomic Features	MRI A total of 1702 radiomic features were extracted from the MRI images (ADC and T2W)	0.71 (0.62-0.79)	0.73 (0.69-0.77)	–	–	ML models using radiomics from ADC maps and T2-weighted images are effective for distinguishing KRAS genes, tumor grading, and EMVI in colorectal cancer.
7	Wesdorp N, Zeeuw M, van der Meulen D, /2023 (24)	Identifying Genetic Mutation Status in Patients with Colorectal Cancer Liver Metastases Using Radiomics-Based Machine-Learning Models.	Portal venous phase CT In total, 851 radiomics features were extracted. Using random forest feature selection, 10 features were selected as the input variables for the classification models. The optimal model parameters were found using cross-validated hyperparameter tuning.	0.86 (0.76-0.95)	0.77	0.93	0.58	Machine-learning models incorporating CT imaging features could identify the genetic mutation status in patients with CRLM with a good accuracy in the internal test set.
8	Porto-Álvarez J, Cernadas E, Aldaz Martínez R/ 2023 (25)	CT-Based Radiomics to Predict KRAS Mutation in CRC Patients Using a Machine Learning Algorithm: A Retrospective Study	abdominal enhancement CT We developed experiments applying the 34 classifiers as input: (1) the clinical vector; (2) the 27 texture feature vectors; (3) combinations of the clinical vector and the 27 texture feature vectors. The texture feature vectors used are: eight Haralick vectors	0.778	0.768	0.733	0.808	Radiomics could help manage CRC patients, and in the future, it could have a crucial role in diagnosing CRC patients ahead of invasive methods.
9	Xue T, Peng H, Chen Q/2022 (26)	Preoperative prediction of KRAS mutation status in colorectal cancer using a CT-based radiomics nomogram	CT 1018 radiomics features were extracted from single-slice and full-volume ROIs	0.93	87% (0.79,0.92)	0.96	0.81	CT-based radiomics showed satisfactory diagnostic significance for the KRAS status in colorectal cancer
10	Cao Y, Zhang G, Bao H /2021 (27)	Development of a dual-energy spectral CT based nomogram for the preoperative discrimination of mutated and wild-type KRAS in patients with colorectal cancer.	monochromatic CT value, iodine concentration, water concentration, and effective atomic number	0.848 (0.779-0.916)	–	0.760;	0.662	Develop nomogram for preoperative discrimination of mutated and wild-type KRAS.
11	Shi R, Chen W, Yang B/ 2020 (28)	Prediction of KRAS, NRAS and BRAF status in colorectal cancer patients with liver metastasis using a deep artificial neural network based on radiomics and semantic features	CT ( portal venous phase) Two semantic and 851 radiomics features were used to predict the mutation status of RAS and BRAF using an artificial neural network method (ANN).	0.95	0.87	0.89	0.84	The application of radiomics together with semantic features can improve non-invasive assessment of the gene mutation status of RAS (KRAS and NRAS) and BRAF in CRLM.
12	Guo XF, Yang WQ, Yang Q/2020 (29)	Feasibility of MRI Radiomics for Predicting KRAS Mutation in Rectal Cancer	MRI 6 features including Glcm-Correlation, Shape-Sphericity, Firstorder-Skewness, Firstorder-Robust Mean Absolute Deviation, Gldm-Large Dependence Low Gray Level Emphasis and Shape-Elongation	0.669	–	0.506	0.773	MRI-based radiomics has the potential in predicting the KRAS status in patients with rectal cancer, which may enhance the diagnostic value of MRI in rectal cancer.
13	Cui Y, Liu H, Ren J,/ 2020 (30)	Development and validation of a MRI-based radiomics signature for prediction of KRAS mutation in rectal cancer	MRI 18 shape features, 14 first-order statistical features, 22 gray-level co-occurrence matrix (GLCM) features, 16 gray-level size zone matrix (GLSZM) features, 16 gray-level run length matrix (GLRLM) features, and 14 gray-level dependence ma-trix (GLDM) features	0.722 (0.654-0.790)	0.681 (0.614-0.743)	0.713 (0.479–0.819)	0.655 (0.504–0.748)	The T2WI-based radiomics signature has a moderate performance to predict KRAS status
14	Wu X, Li Y, Chen X/2020 (31)	Deep Learning Features Improve the Performance of a Radiomics Signature for Predicting KRAS Status in Patients with Colorectal Cancer	Portal venous phase CT 2634 hand-crafted features and 2208 deep learning features were extracted	0.815 (0.766-0.868)	0.746	0.860	0.612	This study presents a model that incorporates the hand-crafted and deep radiomics signature, which can be used for individualized preoperative prediction of KRAS mutations in patients with CRC
15	Oh JE, Kim MJ, Lee J/2020 (32)	Magnetic Resonance-Based Texture Analysis Differentiating KRAS Mutation Status in Rectal Cancer	T2-weighted MR images We extracted the texture features from the T2-weighted MR images (S1 Table). Only three radiomics features were significantly associated with KRASmutation status: Gskewness, SD_ssf_3 and SD_ssf_4.	0.884	0.817	0.81	0.80	T2-weighted images could be used to predict KRAS mutation status preoperatively in patients with rectal cancer.
16	Li Y, Eresen A, Shangguan J, Yang J/2020 (33)	Preoperative prediction of perineural invasion and KRAS mutation in colon cancer using machine learning	Portal venous phase CT Three hundred and six features were extracted from preoperative CT data and important features were determined with a two-step feature selection procedure. 100 variables remained in the feature set of PNI and 106 variables for KRAS mutation.	0.848 (0.819- 0.877)	0.92	–	–	
17	Xu Y, Xu Q, Ma Y/2019 (34)	Characterizing MRI features of rectal cancers with different KRAS status	MRI Mean, Variance, Skewness, Entropy, gray-level nonuniformity, run-length nonuniformity	0.813 (0.746-0.880)		0.7833	0.7449	The MRI findings of rectal cancer, especially texture features, showed an encouraging value for identifying KRAS status.
18	Meng X, Xia W, Xie P/2019 (35)	Preoperative radiomic signature based on multiparametric magnetic resonance imaging for noninvasive evaluation of biological characteristics in rectal cancer	The number of features was 1547. Subsequently, the pair-wise Pearson correlation coefficients were calculated. The threshold for identifying highly correlated feature pairs was 0.9, leaving 427 features for KRAS-2.	0.651(0.539-0.763)	0.616	0.581	0.643	All MRI sequences were important and could provide complementary information in radiomic analysis.
19	Yang L, Dong D, Fang M /2018 (36)	Can CT-based radiomics signature predict KRAS/NRAS/BRAF mutations in colorectal cancer?	shape feature, grey-level histogram feature , GLCM feature, GLRLM feature and the overall feature	0.869 (0.780-0.958)	0.681	0.757	0.833	The proposed CT-based radiomics signature is associated with KRAS/NRAS/BRAF mutations.

## 3 Radiomics work system architecture

In 2012, Lambin et al. first proposed the concept of radiomics (37). Tumors have complex phenotypes and high heterogeneity, which are reflected in different imaging modalities. With the help of radiomics technology (38), multidimensional imaging features can be extracted from medical images in a High throughput manner, and quantitative analysis of the pathophysiological changes in tumors can be carried out, thus enabling an indepth understanding of tumor characteristics. As an emerging interdisciplinary field in the field of precision medicine (39), radiomics has received extensive attention in recent years. Its core goal is to use High throughput feature extraction technology to mine a large number of potential biomarkers from medical imaging data such as CT, MRI, and PET/CT data, facilitating the accurate diagnosis and treatment of diseases (40). The indepth analysis of medical images by radiomics is achieved through the following three key steps:

### 3.1 Multidimensional feature extraction

Preprocessing operations such as denoising, registration, and segmentation are carried out on medical images according to standardized protocols (41). On this basis, multidimensional imaging features are systematically extracted. Morphological features reflect the macroscopic geometric shape of tumors through parameters such as volume and surface area. Texture features analyze the spatial distribution patterns of pixels in images using grey level cooccurrence matrices and run length matrices. Histogram features depict the distribution characteristics of image grayscale values using statistical indicators such as skewness and kurtosis. High order model features capture the complex structures of images using algorithms such as fractal dimension and wavelet transform (42). These high throughput features can comprehensively and meticulously reflect the spatial heterogeneity of tumors (43).

### 3.2 Data standardization and dimensionality reduction

Medical images acquired by different devices vary in imaging parameters, resolution, etc., which can affect the accuracy of the analysis results. Therefore, standardization methods such as Z score, min max normalization, and maximum absolute normalization are used to eliminate device related differences (44). Subsequently, algorithms such as principal component analysis (PCA) and least absolute shrinkage and selection operator (LASSO) are combined to screen high dimensional feature data, remove redundant and irrelevant features and select the key features most relevant to the research objective, laying a solid foundation for subsequent model construction.

### 3.3 Model construction and validation

Using the screened high quality features, various statistical models are applied to further screen the main features closely related to the expected results, thereby improving the accuracy of model prediction. For example, when a model is constructed to associate imaging features with clinical problems, the support vector machine (SVM) can effectively handle nonlinear classification problems and performs well with small sample data (45). A random forest (RF) improves the stability and generalization ability of a model by integrating multiple decision trees and is suitable for high dimensional data (46). The K nearest neighbor algorithm is simple and intuitive, is classified on the basis of the distance between samples, and has no special requirements for data distribution (47). Logistic regression, with its good interpretability, is widely used in disease risk prediction. After model construction, methods such as cross validation and receiver operating characteristic (ROC) curve analysis are also used to evaluate the performance of the model comprehensively to ensure its reliability and effectiveness.

## 4 Progress in predicting KRAS gene mutations in CRC via CT

CT has become the primary examination method for patients with CRC because of its multiple advantages, such as fast imaging speed, high image resolution, multiplanar imaging, high density resolution, and wide application range. The NCCN guidelines also recommend CT as the preferred imaging examination for CRC in clinical practice (48). Currently, CT texture has been used to evaluate the relationship between KRAS mutations and CRC.

On the basis of spectral CT technology, Cao and his research team have carried out indepth explorations on preoperative CRC (27). This team identified multiple spectral CT parameters in the tumor area, providing key evidence for the effective prediction of the KRAS gene mutation status. And discovered the arterial phase slope k, arterial phase effective atomic number, venous phase normalized iodine concentration (NIC), ratio of the enhancement value of the liver segment in the arterial phase to the overall enhancement value of the liver (ATL/LTL ratio), and perfusion fraction index (PFI) are all significant independent predictors of KRAS mutations. Based on these independent predictors, a nomogram model has been developed, with the area under the receiver operating characteristic curve (AUC) reaching 0.848, indicating good calibration performance. It provides a reliable preoperative prediction tool for clinical practice. Yang conducted a retrospective study on 117 CRC patients (36). The researchers systematically extracted 346 radiomics features from the portal venous phase CT images of the patients’ primary tumors. Meanwhile, univariate analysis was analyzed to evaluate the associations between KRAS gene mutations and patients’ clinical background, tumor stage, and histological differentiation. They constructed a radiological feature model using the RELIEFF algorithm and support vector machine, and found a significant correlation between radiomics features and KRAS gene mutation (P<0.001). The model showed good predictive performance in the validation cohort (AUC=0.869), and clinical background, etc., had no significant correlation with KRAS gene mutation (P<0.05), providing an objective imaging basis for clinical practice.

Although certain progress has been made in predicting KRAS gene mutations in CRC patients using CT technology, the field still has limitations such as retrospective studies, small sample sizes, and an inability to replace the gold standard. These limitations indicate that more prospective studies with large sample sizes are needed in the future to promote the further development of CT technology in the field of precisely predicting KRAS gene mutations.

## 5 Progress in predicting KRAS gene mutations in CRC via MRI

MRI has the advantages of multiple parameters, multiple orientations, noninvasive imaging, good tissue contrast, and high spatial resolution and has been widely used in evaluating the KRAS gene mutation status of CRC patients (49, 50). MRI can comprehensively display the lesion site of rectal cancer, providing strong support for clinicians to grasp key information such as the infiltration of the rectal wall in rectal cancer, the metastasis of surrounding lymph nodes, and the status of the circumferential resection margin (51). Currently, it plays an important role in predicting the KRAS gene mutation status of rectal cancer patients (52).

Shin YR reported that KRAS mutation in CRC patients was correlated with N stage (53), the gross morphology of the tumor, the axial length of the tumor, and the ratio of the axial to longitudinal dimensions of the tumor (p = 0.0064, p < 0.0001, p = 0.0003, and p = 0.0090, respectively). The incidence of KRAS mutations was greater in stage N2 (53.70%) and polypoid tumors (59.09%). Tumors with KRAS mutations presented a longer axial length and a greater ratio of axial to longitudinal dimensions. Cui Y et al. conducted a retrospective study, using 400 patients with pathologically diagnosed rectal adenocarcinoma as the training and internal validation sets, and 86 patients from other medical centers as an independent external validation set. They extracted 960 features from T2WI images, performed dimensionality reduction, and constructed models using logistic regression (LR), decision tree (DT), and SVM. The SVM classifier achieved an AUC of 0.714 in the external validation set, indicating that radiomics features from T2WI can assist in predicting KRAS status (30). In addition, the radiomics model constructed by Guo XF and other scholars had an AUC of 0.669 when distinguishing between the KRAS mutation group and the wild type group in CRC patients (29). The radiomics model developed by Xu Y had an AUC ranging from 0.703–0.813 when differentiating between the KRAS wild type group and the KRAS mutation group (54).

Furthermore, the KRAS gene mutation status is important for evaluating the degree of invasion and predicting the prognosis of patients with locally advanced rectal cancer (LARC). Xiang Y constructed a radiomics model for predicting the KRAS gene mutation status by using pretreatment T2WI data and explored the associations among the KRAS gene mutation status, the prediction results of this model, and lung metastasis in detail (21). When predicting KRAS gene mutation in CRC patients, the radiomics model constructed by these researchers achieved AUCs of 0.983 and 0.814 in the training set and validation set, respectively, demonstrating good prediction performance. In addition, this study revealed that patients with a high radiological score (RS) had a greater risk of lung metastasis (HR 3.565, 95% CI 1.337, 9.505, p = 0.011), and the prediction effects were similar for the mutant and wild type KRAS groups (HR 3.225, 95% CI 1.249, 8.323, p = 0.016; IDI: 1.08%, p = 0.687; NRI 2.23%, p = 0.766). In view of this, detecting the KRAS gene status of CRC patients by MRI is indeed a feasible clinical solution (55).

With the rapid development of artificial intelligence technology, some developers have successfully constructed artificial intelligence models that can noninvasively detect the KRAS gene status (56). The detection effect of this model approaches the level of pathological detection, greatly improving the convenience of diagnosis.

## 6 Progress in predicting KRAS gene mutations in CRC via PET/CT

PETimaging is widely used in the diagnosis of CRC, monitoring of treatment response, tracking of disease conditions, and prognosis assessment (57). Fluorodeoxyglucose PET (FDG PET) has a unique advantage. It can automatically generate the contour around the tumor by using quantitative data of glucose uptake within the tumor. Compared with CT and MRI, this technology of automatically outlining the contour significantly reduces the errors caused by observer differences during the image interpretation process, greatly improving the stability and reliability of the diagnostic results.

Among several threshold methods, Chen SW reported that KRAS mutated CRC tumors presented a relatively high SUVmax and increased FDG accumulation. Multivariate analysis revealed that the SUVmax and maximum uptake TW (TW40%) at the 40% threshold level were two predictors of KRAS mutations (58). The odds ratio of SUVmax was 1.23 (P = 0.02; 95% confidence interval was 1.01–1.52), and that of TW40% was 1.15 (P = 0.02; 95% confidence interval was 1.02–1.30). In patients with colon or sigmoid colon cancer, the SUVmax had a greater accuracy in predicting KRAS mutations, whereas in patients with rectal cancer, the accuracy of the SUVmax in predicting KRAS mutations was the same as that of TW40. PET/CT parameters can supplement genomic analysis to determine the expression of KRAS in CRC. This study determined the most effective method to distinguish between mutant and wild type CRC through PET/CT. Kawada K also reported that the accumulation of (18) F-FDG in metastatic CRC was related to the KRAS status. (18) F-FDG PET/CT may help predict the KRAS status of metastatic CRC and contribute to determining a treatment strategy for metastatic CRC (59). Ali MA and others retrospectively analyzed 90 patients with CRC metastases (60). The results revealed that the SUV max, TLG, and TBR of patients with KRAS genotype mutations were significantly greater than those of patients with wild type genotypes. The SUVmax of patients with EGFR exon 20 mutations also increased significantly. Haplotype analysis revealed that the SUVmax of patients with KRAS mutations was significantly greater than that of other patients, with a specificity of 68.18% and a sensitivity of 65.28%. The research results indicate that the [18F] FDG PET/CT radiological parameters, especially the SUV max, have the potential to serve as noninvasive tools for predicting the KRAS/BRAF/EGFR mutation status of mCRC patients. Zhao H et al. extracted radiomics features from the entire tumor region, tumor habitat based radiomics features, and metabolic parameters from 18F-FDG PET images. After dimensionality reduction, a hierarchical model for predicting KRAS mutation status in CRC patients was constructed using the support vector machine algorithm. The results showed that the model had strong predictive ability, with an AUC of 0.701 in the validation group. Shapley additive explanation analysis indicated that the tumor microenvironment and hypermetabolic regions had the most significant impact on the model’s prediction results (20).

Decision making Therefore, we believe that the radiomics model constructed based on PET/CT can analyze the characteristics of CRC tumors from multiple dimensions, excavate information such as metabolism and morphology, and predict the therapeutic response of CRC. It provides an objective and comprehensive basis for clinicians to formulate adjuvant treatment plans for CRC, which is more accurate and less risky than traditional methods, and has broad promotion prospects.

## 7 Existing challenges and future prospects

### 7.1 Current technical challenges in multimodal imaging and radiomics

Although there have been phased achievements in the detection of the KRAS gene mutation status in CRC patients, the current field still faces many technical problems and challenges in clinical translation. Most of the current research is limited to a single imaging modality, such as CT, MRI, or PET/CT, and indepth fusion analysis of multimodal imaging has not been carried out. In fact, MRI has excellent resolution for soft tissues, CT can provide accurate anatomical positioning, and PET/CT can effectively reflect metabolic activity. After these three methods are integrated, a multidimensional feature space of “structure function metabolism” can be constructed, providing more comprehensive information for clinical detection. Therefore, we recommend committing to the development of an innovative deep fusion artificial intelligence analysis framework for multimodal imaging. This framework is designed with dual advantages: it can not only perform independent feature extraction and dimensionality reduction analysis on single modality images such as CT, MR, and PET images but also achieve collaborative fusion and joint modelling of multimodal data. Technically, the core value of multimodal imaging lies in its ability to integrate the complementary advantages of different imaging techniques, including the excellent density resolution of CT, the superior soft tissue contrast of MR, and the unique metabolic activity information of PET (59). The fusion of this multidimensional information is expected to significantly increase diagnostic efficiency (20).

However, issues such as spatiotemporal alignment, feature standardization, and weight distribution among different imaging modalities still remain key technical problems hindering the application of multimodal imaging. Cross deviceCross device But first order features, geometric morphological features, and general features derived from deep learning are less dependent on inter machine variability. First order features, based on basic statistical properties of pixel values such as mean, variance, and histogram distribution, are suitable for preliminary feature extraction of Cross device and cross modal data to reduce feature bias caused by equipment differences. Geometric morphological features describe the morphological attributes of lesions or tissues, such as volume, surface area, diameter, lobulation sign, and spiculation sign. These features, relying on regional analysis after image segmentation, can partially transcend inter machine differences. General features derived from deep learning are high level semantic features extracted from images using pretrained models. If the model undergoes transfer learning on Cross device datasets, its feature representation can have a certain degree of device invariance. Therefore, in the application of multimodal imaging, first order statistical and geometric morphological features can serve as the basis for Cross device feature analysis due to their low dependence on inter machine variability, while deep learning features require data augmentation and transfer learning to optimize their generalizability. The heterogeneity of clinical imaging data, equipment differences, and the lack of unified standards pose challenges to detection work, which require further exploration in future research.

### 7.2 Standardized quality assessment (radiomics quality score and quality assessment of diagnostic accuracy studies-2)

The reproducibility and clinical translation value of radiomics research highly depend on the rigor of the methodology. Therefore, we recommend the use of the Radiomics Quality Score (RQS) to systematically evaluate study design, image analysis, model construction, and clinical applicability (61). RQS is a widely recognized quality assessment tool in the radiomics field, comprising 16 scoring items (total score 36 points) across the following key dimensions: 1. Image acquisition and preprocessing (maximum 5 points); 2. Feature Extraction and Stability (maximum 9 points); 3. Model Construction and Validation (maximum 12 points); 4. Reporting transparency and open science (maximum of 5 points); 5. Clinical relevance (maximum 5 points). RQS enables systematic evaluation of the complete quality chain in radiomics research and provides clear directions for improvement in subsequent studies.

The Quality Assessment of Diagnostic Accuracy Studies-2 (QUADAS-2) is an internationally authoritative quality assessment tool for diagnostic accuracy studies proposed by Whiting et al. in 2011 (62). It addresses the problem of inconsistent standards for evaluating diagnostic tests, helps researchers identify bias risks and validate the clinical applicability of diagnostic methods, and provides a data foundation for evidence based medicine. QUADAS-2 establishes a rigorous and user friendly evaluation framework covering four core domains: case selection, index of interest, reference standard, and flow timing. For each domain, assessments are conducted for both “risk of bias” and “applicability,” yielding three types of judgements: low, high, or unclear. Standardized evaluation using QUADAS-2 effectively mitigates common biases in diagnostic accuracy research. For example, case selection bias may lead to overestimation or underestimation of a diagnostic method’s accuracy, whereas verification bias (where some patients receive only the index test without reference standard validation) can introduce systematic deviations. QUADAS-2 enhances the reliability and robustness of results, clarifies the clinical applicability of diagnostic models, and provides an objective basis for clinicians to interpret results and select methods.

### 7.3 Limitations of existing machine learning models

In addition, most existing machine learning models are trained on single center data, resulting in poor universality of the models. When cross institutional verification is conducted, the AUC value is likely to decrease. Owing to the “black box” characteristic of deep learning models, they cannot meet the clinical requirements for the interpretability of Decision making bases. Especially in scenarios where the KRAS status is closely related to the treatment plan, doctors need to clarify the causal relationship between imaging features and KRAS gene mutations, and “black box” models have difficulty providing support. This will make it impossible to determine whether the model’s judgements are based on genuine biological characteristics or data bias and make establishing pathophysiological correlations between radiomics features and KRAS mutations difficult; moreover, it fails to comply with the “interpretability” requirements for medical AI product registration (such as relevant regulations from the FDA and NMPA).

### 7.4 Future directions for technological breakthroughs and solutions

To overcome the above bottlenecks and promote the development of detection technology, the following aspects can be considered in the future: first, establish a multicenter imaging database; second, implement standardized scanning protocols, such as the MRI scanning protocol for CRC recommended by the American Society of Neuroradiology (ASNR); and third, formulate a standardized process for radiomics feature extraction. Standardize feature naming, calculation methods, and quality control to lay a solid data foundation. Among them, the standardization and update of the general lexicon of radiomics can be achieved through consensus on term definitions by multidisciplinary teams and the public and dynamic maintenance of the terminology database (establishing a dynamically updated online dictionary, such as RadLex) and incorporating feature codes, reference values and application scenarios. Second, radiological features with high Cross device consistency and repeatability should be extracted and adopted as much as possible, such as first order statistical features, morphological features, anatomical structure features, quantitative biomarkers, features based on relative measurements, high contrast regional features, and global features that are insensitive to local noise or small regional artefacts. Third, cross modal feature alignment algorithms, such as the generative adversarial network (GAN), should be developed to achieve modality conversion and eliminate the impact of equipment differences on imaging data. By constructing a hierarchical feature fusion model, starting from the integration of original pixel features at the bottom layer to the fusion of semantic features at the middle layer and finally outputting the prediction results at the top layer, the accuracy and clinical practicality of the model’s prediction results can be ensured. Furthermore, a visual Decision making path was constructed to clarify the correlation strength between radiomics features and KRAS mutations. An end to end model is built to automate feature extraction and model construction and establish a fully automatic intelligent push system to provide a visual operation interface for clinicians, facilitating the widespread application of artificial intelligence models in clinical scenarios.
